# Dietary Fiber Lacks a Consistent Effect on Immune Checkpoint Blockade Efficacy Across Diverse Murine Tumor Models

**DOI:** 10.1158/0008-5472.CAN-24-4378

**Published:** 2025-06-20

**Authors:** Asael Roichman, Gabriela Reyes-Castellanos, Ziqing Chen, Zihong Chen, Sarah J. Mitchell, Michael R. MacArthur, Akshada Sawant, Llewelyn Levett, Jesse R. Powers, Victoria Burgo, Maria Gomez-Jenkins, Maria Ibrahim, Xincheng Xu, Beianka Tomlinson, Xiang Hang, Eric G. Pamer, Yong Wei, Yibin Kang, Eileen P. White, Joshua D. Rabinowitz

**Affiliations:** 1 Department of Chemistry, Princeton University, Princeton, New Jersey.; 2 Lewis-Sigler Institute of Integrative Genomics, Princeton University, Princeton, New Jersey.; 3 Ludwig Institute for Cancer Research, Princeton Branch, Princeton, New Jersey.; 4 Department of Molecular Biology and Biochemistry, Rutgers University, New Jersey.; 5 Rutgers Cancer Institute, New Brunswick, New Jersey.; 6 Department of Molecular Biology, Princeton University, Princeton, New Jersey.; 7 Duchossois Family Institute (DFI), University of Chicago, Chicago, Illinois.

## Abstract

Immune checkpoint blockade (ICB) has transformed cancer treatment, but success rates remain limited. Recent research suggests that dietary fiber enhances ICB efficacy through microbiome-dependent mechanisms. However, prior studies in mice compared grain-based chow (high fiber) with low–fiber-purified diet, but these diets also differed in other dimensions, including phytochemicals. Therefore, further work is needed to establish the robustness of the effect of fiber on ICB across cancer types and dietary contexts. In this study, we investigated gut microbiome composition, metabolite levels, and ICB activity in mice fed with grain-based chow or purified diets with differing quantities of isolated fibers (cellulose and inulin). Compared with dietary fiber content, consumption of chow versus purified diet had a greater effect on the gut microbiome and a much stronger impact on the metabolome. Studies in multiple tumor models revealed that fiber has a weaker impact on ICB (anti–PD-1) efficacy than previously reported. Although diet affected ICB in some models, the effect was not directionally consistent. None of the models tested displayed the pattern expected if fiber controlled ICB efficacy: strong efficacy in both chow and high–fiber-purified diet but low efficacy in low–fiber-purified diet. Thus, dietary fiber seems to have limited or inconsistent effects on ICB efficacy in mouse models, and other dietary factors that correlate with fiber intake may underlie clinical correlations between fiber consumption and immunotherapy efficacy.

**Significance:**

Clinical associations between high-fiber diets and improved immunotherapy efficacy may be driven by dietary factors correlated with fiber intake rather than fiber itself, which could impact dietary recommendations for patients undergoing immunotherapy.

## Graphical Abstract

**Figure:**
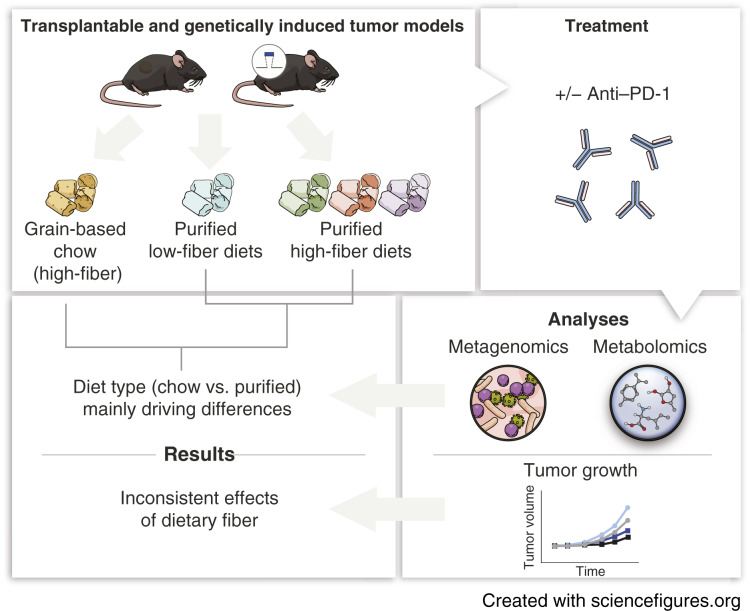

## Introduction

The development of immune checkpoint blockade (ICB)–based immunotherapy has transformed the field of cancer therapy, giving hope for cure in conditions previously considered untreatable. However, despite this significant advancement, response to ICB therapy varies, ranging from approximately 45% to 60% in melanoma to only 15% to 30% in most solid tumors (1, 2). Factors associated with favorable response to ICB include DNA mismatch repair deficiency, microsatellite instability, high tumor mutational burden, and high PD-L1 expression (3, 4). These factors are not, however, under the control of the patient. A modifiable factor that is increasingly showing promise to predict responses to ICB in both mice and humans is the gut microbiome (3, 4). The gut microbiome—the collection of microorganisms colonizing the colon—plays a major role in antitumor immunity, with diverse gut microbiome compositions associated with favorable anticancer response to ICB (5–7). Interventions designed to modify the microbiome into a more favorable composition may therefore have the potential to enhance response rates to ICB therapy.

One way to manipulate microbiome composition is by introducing beneficial bacteria directly through a probiotic cocktail or by performing fecal microbiome transplant (FMT) of whole microbial communities from donors with desirable microbial compositions. FMT from ICB responders has shown promise in some patients with melanoma for improving response to ICB, whereas other patients did not respond favorably (8). One possible reason for treatment failure is that microbes delivered by FMT may not efficiently colonize the recipient’s gut, thereby failing to induce the desired changes in microbiome compositions (8).

An alternative or complementary way to manipulate gut microbiome composition and potentially improve ICB response is through dietary changes (9, 10). A key dietary ingredient that feeds the gut microbiome and can be therefore used to influence its composition is dietary fiber (10, 11). Dietary fibers are edible carbohydrate polymers that are resistant to endogenous digestion in the small intestine. They can be classified into two categories: (i) insoluble fibers, such as cellulose, hemicellulose, and lignin, which are slowly or minimally digested by the gut bacteria and (ii) soluble fibers, such as inulin, pectin, and β-glucans, which are readily metabolized by the gut microbiota (11). Spencer and colleagues (12) recently investigated the role of dietary fiber in the response to ICB. Interestingly, the study found that patients with melanoma who consume sufficient fiber (evaluated based on a dietary questionnaire) demonstrated improved progression-free survival rate relative to patients with insufficient fiber intake.

As causality could not be inferred from this observational human cohort, results were validated in mice. Specifically, in transplantable melanoma and MC-38 colon carcinoma models, specific pathogen-free C57BL/6 mice consuming a grain-based chow, which is high in fiber, showed improved response to ICB (anti–PD-1 or anti–PD-L1) relative to mice fed a diet low in fiber (12). Motivated by these results, clinical trials testing the effect of high-fiber diet on ICB outcomes are ongoing (e.g., NCT04645680, NCT06298734, and NCT04866810).

When conducting dietary intervention studies, it is critical to account for various aspects of the diets. In their mouse studies, Spencer and colleagues (12) investigated the effect of fiber by comparing a grain-based rodent chow as the high-fiber diet (17.6% total fiber) with a purified ingredient low-fiber diet (8% cellulose with no soluble fiber). Notably, grain-based chows and purified diets are inherently different: grain-based chows are manufactured primarily from unrefined ingredients (typically ground corn, soy, wheat, and fish), whereas purified diets are prepared from refined, purified ingredients (e.g., casein protein, oils, and starch; ref. 13). In terms of fiber, standard grain-based chows typically contain 15% to 20% fiber comprising a complex mixture of soluble and insoluble fibers, whereas purified diets contain specific fibers, typically cellulose (insoluble) and inulin (soluble). As such, chow and purified diets differ not only in fiber composition but also in multiple other factors including the source of macronutrients, vitamins, minerals, and the presence of phytochemicals (plant secondary metabolites) and heavy metals (13). Any of these factors could potentially contribute to the observed synergy between chow and ICB therapy. To conclusively determine the role of fiber and identify the types that may be most effective, it is necessary to compare diets that vary in fiber content but are otherwise identical.

In this study, we explored the effect of dietary fiber on ICB efficacy by feeding mice either typical grain-based chow or purified diets with varying fiber (high/low cellulose/inulin). We used five preclinical tumor models representing varied cancer types and conducted the experiments across different animal facilities. Our key questions were: (i) whether fiber is the primary cause of gut microbiome and metabolic differences between grain-based chow and purified diets, (ii) whether grain-based chow consistently enhances response to ICB therapy relative to low–fiber-purified diet, and (iii) whether dietary fibers improve ICB efficacy when tested within a controlled purified diet, and if so, whether specific types of fibers, such as insoluble versus soluble fibers, are more effective than others. Overall, we find that chow versus purified diet has massive impact on the circulating metabolome largely independent of fiber and that different diets can affect ICB efficacy in different tumor models, but with no consistent pattern observed across models and no consistent trend toward high fiber being desirable.

## Materials and Methods

### Mice and diets

All mouse studies were performed in accordance with Institutional Animal Care and Use Committee–approved animal protocols at Princeton University (PU), Rutgers Cancer Institute of New Jersey, and at Charles River Laboratories (CRL). C57BL/6 mice were purchased from CRL (#027, RRID: IMSR_CRL:027) for MC-38 experiment (females) or from The Jackson Laboratory (#000664, RRID: IMSR_JAX:000664) for B16-OVA (females) and YUMM1.1-9 (males) experiments and were 6 to 10 weeks old at the date of diet switch. Female FVB/N-Tg(MMTV-PyVT)634Mul/J (#002374, RRID: IMSR_JAX:002,374) mice were purchased from The Jackson Laboratory at 6 to 7 weeks of age. After 1 to 2 weeks of acclimation to the facility, mice were switched to one of three experimental diets (Supplementary Table S1). During the acclimation period, mice were fed chow (PicoLab Rodent Diet 5053, LabDiet).

The DNA polymerase delta 1 (*Pold1*) mouse model was created at the Rutgers Cancer Institute together with the Genome Editing Shared Resource. Using CRISPR/Cas9 technology, a point mutation (D400A) was created in the proofreading domain of DNA polymerase δ as described (bioRxiv 2024.06.10.597960). Mice on a C57BL/6 background were used to generate the *Pold1* mouse model. Briefly, *Pold1*^+/+^ mice were crossed with *Pold1*^*+/D400A*^ to obtain heterozygous mice. Then, *Pold1*^*+/D400A*^ × *Pold1*^*+/D400A*^ breeding was used to obtain *Pold1*^*D400A/D400A*^ homozygous mutants. *Pold1*^*D400A/D400A*^ homozygous mice were bred to get experimental mice. The progeny from homozygous breeding was not used for breeding to avoid accumulation of mutations over the generations. PCR amplification and DNA sequencing were used to verify the mutant genotype, and only homozygous mice were used in this experiment. At an average of 2 months old, mice were switched from chow diet (PicoLab Rodent Diet 5058) to the LC_In0 or HC_In0 diets.

Body weight and food intake were monitored once or twice per week. During all points of the studies, mice had free access to food and water *ad libitum*, except PyMT experiment in which the mice were overnight fasted before the day of diet switch. Mice were maintained on a 12-hour light–dark cycle.

### Tumor inoculation and endpoints

For transplantable models, 1 week prior to inoculation, mice were switched to one of the experimental diets (Supplementary Table S1). On day 7, mice were injected subcutaneously with 2.5 × 10^5^ B16-OVA cells (generously provided by Dr. Marcia Haigis and Dr. Arlene Sharpe), 5 × 10^5^ MC-38 cells (RRID: CVCL_B288, generously provided by Dr. Arlene Sharpe), or 1 × 10^6^ Yumm1.1-9 cells resuspended in 0.1 mL PBS. Yumm1.1 cells (RRID: CVCL_JK10) were obtained from the Bosenberg lab (Yale University) and UV irradiated to generate Yumm1.1-9 cells as described (14). Cell lines were authenticated by the source and checked in laboratory for appropriate morphology and were periodically tested for *Mycoplasma*. Cells were cultured for a maximum of 3 weeks before inoculations. Mice were followed twice a week with body weight and tumor volume measurements. Mice were euthanized by CO_2_ asphyxiation if they reached the endpoints: body weight loss of >20% of peak body weight, body condition score of ≤1.5, inappetence/dehydration, tumor diameter >2.0 cm, tumor volume >2.0 cm^3^, tumor burden >10% of body weight, ulceration of tumor or self-mutilation, inability to access food or water, or breathing problems/cyanosis. Tumor volume (mm^3^) was calculated by the formula (length × width × height) × 0.5 (MC-38 in PU and B16-OVA) or by (length × width^2^) × 0.5 (MC-38 in CRL, YUMM1.1-9, and PyMT) using digital calipers (Thermo Fisher Scientific). Lung nodules (PyMT model) were counted directly after fixation in Bouin solution (HT101128, Sigma). The lung images were taken using a Zeiss SteREO Discovery microscope. For the *Pold1* model, necropsy with tissue sampling was performed to confirm the presence of cancer when the mice reached ethical endpoint.

### Anti–PD-1 or isotype control treatment

For transplantable models, mice were randomized and assigned to treatment groups when the average tumor volume reached 40 to 100 mm^3^. For MC-38 at CRL, mice were treated intraperitoneally with anti–PD-1 (ICH1132, clone RMP1-14, ichorbio, RRID: AB_11203476) or isotype control (BE0089, rat IgG2a, Bio X Cell, RRID: AB_1107769) at 5 mg/kg twice per week for 2 weeks. For MC-38 at PU and B16-OVA, mice were dosed with 100 μg of anti–PD-1 (BE0273, clone 29F.1A12, Bio X Cell, RRID: AB_2687796) or isotype control (BE0089, rat IgG2a, Bio X Cell, RRID: AB_1107769). This was administered as a 100 μL intraperitoneal injection diluted in *InVivo*Pure pH 7.0 dilution buffer (IP0070, Bio X Cell) for anti–PD-1 or *InVivo*Pure pH 6.5 dilution buffer (IP0065, Bio X Cell) for the IgG2a isotype control. Mice were dosed twice per week for 2 weeks for MC-38 and every 3 days for a total of eight dosages for B16-OVA. For YUMM1.1-9, mice were treated with anti–PD-1 antibody (ICH1132, clone RMP1-14, ichorbio, RRID: AB_11203476) or isotype control (ICH2244, rat IgG2a, Ichorbio, RRID: AB_2921379) at 10 mg/kg (both diluted in PBS) every 3 days for a total of eight dosages. For PyMT, mice were randomized and diets were switched at 9 weeks of age, and anti–PD-1 treatment at 10 mg/kg was given at weeks 10 to 15, twice per week (week 10–12, clone BE0146 and clone RMP1-14, Bio X Cell, RRID: AB_10949053; week 13–15, BE0273, clone 29F.1A12, Bio X Cell, RRID: AB_2687796). For the *Pold1* model, 1 week after changing diets, mice were randomly assigned to anti–PD-1 or isotype groups and treatments were initiated and administered every other week. Mice were injected intraperitoneally with 250 μg/mouse of anti–PD-1 antibody (BE0146, clone RMP1–14, Bio X Cell, RRID: AB_10949053) or isotype (BE0089, rat IgG2a, Bio X Cell, RRID: AB_1107769) diluted in 100 μL of PBS.

### Serum and feces sampling

Blood was collected by tail snip in *ad lib* fed mice on the following time points: for B16-OVA, around 9:00 am the day after the sixth dose of anti–PD-1 or isotype control, for YUMM1.1-9, between 6 and 8:00 am the day after the third dose of anti–PD-1 or isotype control, and for PyMT, between 2 and 3:00 pm the day after the fifth dose of anti–PD-1 or isotype control. Blood was centrifuged at 10,000 *g* at 4°C for 10 minutes. Serum was isolated from the supernatant and stored at −80°C until further analysis. Feces samples were collected at the same time window as for blood sampling and stored at −80°C until further analysis.

### Fecal DNA extraction

Fecal samples for metagenomic analysis were collected from B16-OVA tumor-bearing mice the day after the sixth dose of anti–PD-1 or isotype control treatment. DNA from samples was extracted using the QIAamp PowerFecal Pro DNA kit (Qiagen). Prior to extraction, samples were subjected to mechanical disruption using a bead beating method. Briefly, the samples were suspended in a bead tube (Qiagen) along with lysis buffer and loaded on a bead mill homogenizer (Qiagen, TissueLyser II). The samples were then centrifuged, and the supernatant was resuspended in a reagent that effectively removes inhibitors by precipitating non-DNA organic and inorganic materials to increase DNA purity. DNA was then purified using a silica spin column filter membrane. DNA was bound selectively to the membrane by adding a high-concentration salt solution, which was then eluted using elution buffer. DNA was quantified using Qubit.

### Shotgun metagenomics

Libraries were prepared using 200 ng of genomic DNA using the QIAseq FX DNA Library Prep Kit (Qiagen). For samples with ultra-low DNA concentration (10 ng or less), the FX enhancer reagent was used. DNA was fragmented enzymatically using a nuclease and desired insert size was achieved by adjusting fragmentation conditions. Fragmented DNA was end repaired using end repair enzyme and “A”s were added to the 3′ ends to stage inserts for ligation. During ligation, Illumina-compatible unique dual index adapters were attached to the inserts and the prepared library was PCR amplified. Amplified libraries were purified, and quality control was performed using a TapeStation 4200 (Agilent). Normalized libraries were prepared as paired-end libraries with insert size around 350 bp for each sample. High-throughput sequencing on Illumina NextSeq 2000 produced 11 to 16 million paired-end reads per sample with a read length of 150 bp.

### Metagenomic data processing

Adapters were trimmed off from the raw reads, their quality was assessed and controlled using Trimmomatic (v.0.39; ref. 15), and then the human genome was identified and removed by kneaddata (v0.7.10, https://github.com/biobakery/kneaddata). Taxonomic classification of the resultant high-quality reads was run against Kraken2 (v. 2.1.3, database dated: 2024/05/01; ref. 16), normalized to total classified reads, and relative abundance was tabulated. Microbial reads were assembled using MEGAHIT (v1.2.9; ref. 17) and annotated with Prodigal (v2.6.3; ref. 18). Count data were analyzed in R using the phyloseq package (v1.50.0; ref. 19). Principal coordinate analysis was conducted using the Bray–Curtis dissimilarity index. Differential abundance analysis was performed using the edgeR package (v 4.4.2; ref. 20), and heatmaps were generated using MicrobiomeAnalyst (v 2.0; ref. 21) and the pheatmap package (v1.0.12; https://cran.r-project.org/web/packages/pheatmap/index.html, RRID: SCR_016418).

### Metabolite extraction

For serum samples from B16-OVA, YUMM1.1-9, and PyMT studies, 2.5 μL of serum was added into 80 μL of ice-cold methanol and stored in −80°C for at least 30 minutes. Samples were centrifuged at maximum speed (21,380 *g*) at 4°C for 20 minutes. Supernatants were collected for LC-MS analysis. For feces samples from B16-OVA and YUMM1.1-9 studies, frozen feces samples were transferred to Eppendorf tubes with ceramic beads on dry ice and disrupted by CryoMill (Retsch). About 10 mg of homogenized powder was weighed and extracted by ice-cold acetonitrile:methanol:water (40:40:20, supplemented with 0.5% vol formic acid). Extracts were vortexed for 10 seconds, kept on dry ice for 10 minutes, and neutralized by NH_4_HCO_3_ (15% in water, 8.8% v/v of extraction buffer was used). Neutralized extracts were vortexed for 10 seconds again, kept on dry ice for 1 hour, and centrifuged at 21,380 *g* at 4°C for 20 minutes. Supernatants were collected for LC-MS analysis.

### LC-MS method

Metabolites were separated by hydrophilic interaction liquid chromatography with an XBridge BEH Amide column (2.1 mm × 150 mm, 2.5 μm particle size; Waters, 186006724). The column temperature was set at 25°C. Solvent A was 95 vol% H_2_O and 5 vol% acetonitrile (with 20 mmol/L ammonium acetate and 20 mmol/L ammonium hydroxide, pH 9.4). Solvent B was acetonitrile. The flow rate was 0.15 mL/minute. The liquid chromatography gradient was 0 to 2 minutes, 90% B; 3 to 7 minutes, 75% B; 8 to 9 minutes, 70% B; 10 to 12 minutes, 50% B; 13 to 14 minutes, 25% B; 16 to 20.5 minutes, 0.5% B; and 21 to 25 minutes, 90% B. Mass spectrometry analysis was performed on Thermo Fisher Q Exactive Plus (QE+) Hybrid Quadrupole-Orbitrap, Orbitrap Exploris 240, or Orbitrap Exploris 480 mass spectrometer, with injection volume of 5 to 10 μL. Two scans were performed, one in negative mode (m/z of 70–1,000) and one in positive mode (m/z of 119–1,000). Other parameters for QE+ were resolution 140,000 for negative mode and 70,000 for positive mode, AGC target 3e6, and max IT 300 ms. For Exploris 240, resolution 120,000, AGC target 1,000% for negative mode and 500% for positive mode, and max IT 500 ms. For Exploris 480, resolution 120,000, AGC target 1,000%, and max IT 500 ms.

### Immune profiling, sample preparation, and flow cytometry

For immune profiling in the PyMT model, 0.5 g primary tumor was harvested and dissected in 1.5-mL tubes prior to digestion with the Tumor Dissociation Kit (130-096-730, Miltenyi Biotec). Tumors were then dissociated to single-cell suspensions according to the manufacturer’s instruction. Cells were spun down at 500 g for 5 minutes, and the supernatant was removed and then resuspended in 3 mL ACK buffer for red blood cell lysis. The cells were briefly vortexed, allowed to sit at room temperature for 1 to 3 minutes, and then quenched with 10 mL of Dulbecco’s PBS. The cells were then spun down and resuspended in FACS buffer (Dulbecco’s PBS containing 2% FBS) for experiment. Single-cell suspensions were first incubated with anti–mouse FcγIII/II (CD16/CD32) receptor blocking antibodies (Thermo Fisher Scientific) for 10 minutes, followed by staining with antibodies (Supplementary Table S2) for 30 minutes at 4°C. Fixable viability dye Near IR (Thermo Fisher Scientific) or Aqua (Thermo Fisher Scientific) was used to exclude dead cells. All data were collected on an Attune NxT Flow Cytometer and analyzed with FlowJo 10.7.1. Gating for experiments is shown in Supplementary Fig. S1.

### Data and statistical analysis

**Figure 1.**
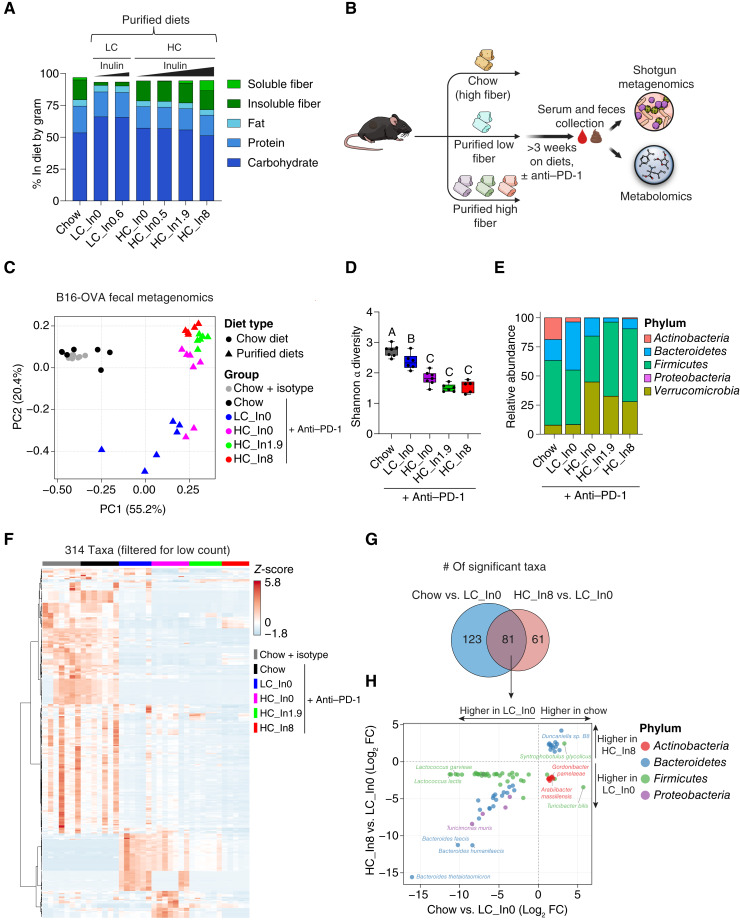
Microbiome composition is primarily shaped by diet type and is partially restored to chow-like levels by isolated fiber. **A,** Macronutrient and fiber composition of diets used in this study represented by %gram. In each tumor experiment, we used one of the low cellulose (LC)–purified diets (low fiber) and up to four of the high cellulose (HC)–purified diets (high fiber). LC = 2.5 to 2.6 %gram and HC = 14.9 to 15.7 %gram. Numbers in diet names represent %gram of inulin (In) in the diets. **B,** Schematic of the study design for the metagenomics (this figure) and metabolomics (Fig. 2). **C,** Principal coordinate analysis of all metagenomic samples from the B16-OVA experiment. PC, principal component. **D,** Shannon α diversity index across the dietary groups. Boxes represent the IQR with median shown as a horizontal line, whiskers extend from minimum to maximum values, and different letters above the bars indicate statistically significant differences between groups (*P* < 0.05) by a one-way ANOVA followed by the Tukey *post hoc* test. **E,** Relative abundances of microbiome composition at the phylum level, shown as the mean for each group. **F,** Heatmap depicting the relative abundances of 314 taxa after filtering out low-abundance taxa (<30 counts in >85% of samples). **G,** Venn diagram of significantly changed taxa in mice fed with chow vs. low–fiber-purified diet (LC_In0) and in mice fed with high–fiber (HC_In8)- vs. low–fiber (LC_In0)-purified diet (all B16-OVA tumors and anti–PD-1 treatment). **H,** Scatter plot of the 81 taxa altered by both chow and high-fiber–purified diet, displaying correlations between these two conditions. Each dot represents a specific taxon, with dot color indicating the taxon’s phylum as specified in the legend. For **C**–**H**, data are from *n* = 5 to 7 mice per group. FC, fold change. (**B,** Created with sciencefigures.org.)

Raw data were collated in Microsoft Excel before being transferred to GraphPad Prism or R (for metagenomics) for analysis. The heatmap in Fig. 1F was generated using MicrobiomeAnalyst 2.0 (https://www.microbiomeanalyst.ca/). Metabolomics data were analyzed using MetaboAnalyst 5.0 (https://www.metaboanalyst.ca/). Data are presented as mean ± SEM for bar and line graphs. For multiple-group comparisons, we used one-way or two-way ANOVA with Šídák or Tukey multiple comparison correction. Survival was analyzed by Kaplan–Meier survival curves, and *P* values were calculated by a log-rank or Wilcoxon test using GraphPad Prism and corrected for multiple comparisons by the Benjamini–Hochberg method. All comparisons were two-tailed, and a *P* value below 0.05 was considered a statistically significant difference. Significant outliers determined by the Grubb test were removed from final analysis. For metagenomic analysis, taxa with FDR <0.01 and |fold change| >2 were considered statistically significant.

### Data availability

Shotgun metagenomics sequencing data are available on NCBI under BioProject PRJNA1235712. Metabolomics data were deposited to Metabolomics Workbench and are available under the study identifiers ST003789 and ST003790. All raw data generated in this study are available upon request from the corresponding authors.

## Results

### Diet design

To study the impact of dietary fiber on response to ICB, we used a grain-based chow diet, which contains a complex fiber mixture (∼15.5% w/w insoluble fiber and ∼1.9% w/w soluble), and designed six purified diets varying in their fiber content, with cellulose added as insoluble fiber and inulin as soluble fiber. The six purified diets closely matched to chow in macronutrient composition (Supplementary Fig. S2A). Two diets were low in total fiber, containing low cellulose (LC; 2.6% w/w) and either no or low amount of inulin (0.6% w/w). Four diets had high fiber, containing high cellulose (HC; ∼15.5% w/w, designed to match the amount of insoluble fiber in chow) and variable amounts of inulin (In, 0%–8% w/w; Fig. 1A, see also Supplementary Table S1). The diet with HC and intermediate inulin (1.9%) was designed to contain both insoluble and soluble fibers at similar levels to chow.

### Fiber partially accounts for gut microbiome differences between purified diet and chow

We first sought to determine the impact of grain-based chow and fiber supplementation in purified diets on the gut microbiome. To this end, we conducted shotgun metagenomics on feces of mice fed with chow, low–fiber-purified diet, or one of three high–fiber-purified diets. To ensure relevance to the anti–PD-1 efficacy experiments, metagenomic analysis was performed on B16-OVA melanoma tumor–bearing mice treated with either isotype control or anti–PD-1 (Fig. 1B). We first assessed the differences in community composition between all samples by performing principal coordinate analysis. Samples were clustered by diet with diet type (chow vs. purified) as the primary driver of variation (first principal coordinate) and fiber content as a secondary driver (Fig. 1C). Consistent with the notion that complex diets promote microbial diversification (22), within-sample α diversity was lower in purified diets compared with chow. Insoluble fiber (HC) decreased rather than increased diversity, suggesting that microbiome diversity arises from diet complexity rather than fiber *per se* (Fig. 1D).

Phylum-level analysis of microbial composition revealed that the high–fiber-purified diets led to increased abundance of *Verrucomicrobia* (largely *Akkermansia*) and reduced levels of *Actinobacteria*, as well as *Bacteroidetes* and *Firmicutes* profiles that more closely resembled chow-fed animals (Fig. 1E; Supplementary Fig. S2B). We next assessed microbiome composition at the taxonomic level (Supplementary Table S3). Hierarchical clustering of microbial taxa was consistent with the community-level analysis, with differences mainly driven by diet type, followed by fiber levels in purified diet (Fig. 1F; Supplementary Table S4). Relative to low–fiber-purified diet, 204 taxa were significantly changed in chow and 142 in HC with 8% inulin high–fiber-purified diet. A majority of the 81 taxa changed by fiber overlapped with those changed by chow (Fig. 1G; but not *vice versa*), with chow and fiber having the same direction of effect in most cases (86%; Supplementary Fig. S2C and S2D). Among the most strongly affected taxa, *Bacteroides thetaiotaomicron* was low in both chow and high–fiber-purified diets (HC with either 1.9% or 8% inulin), whereas *Duncaniella sp. B8* was higher in both chow and high–fiber-purified diets (Supplementary Fig. S2D and S2E). Moreover, the abundance of taxa from the *Bacteroidetes* phylum was correlated between chow and the 8% inulin high–fiber-purified diet (Fig. 1H). Species previously linked to improved clinical responses to ICB include *Akkermansia muciniphila* (23) and *Faecalibacterium prausnitzii* (12, 23)*.* The former was high with HC-purified diet and the latter with chow (Supplementary Fig. S2F). Thus, fiber and chow induce different ICB-favoring bacteria. Collectively, these findings suggest that isolated fiber partially recapitulates the microbial compositional changes induced by chow, with many other microbiome changes related instead to dietary complexity (i.e., chow specific).

### Grain-based chow and purified diets induce fundamental metabolomic differences independent of fiber content

**Figure 2.**
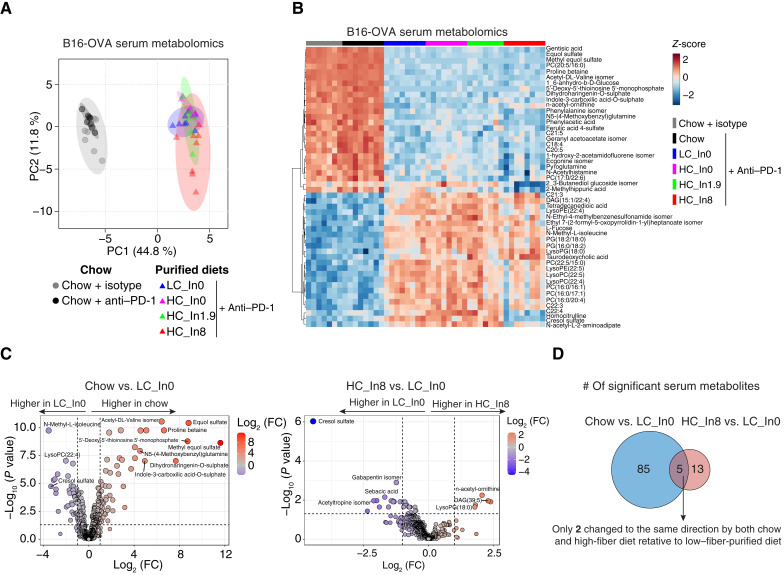
Purified diet has a greater impact than fiber on the serum and fecal metabolome. **A,** PCA of serum metabolomics from the B16-OVA experiment. PC, principal component. **B,** Heatmap showing log-transformed relative abundances of top 50 significant metabolites by ANOVA of the same metabolomics dataset. **C,** Volcano plots of serum metabolites in mice fed with chow vs. low–fiber-purified diet (LC_In0) and in mice fed with high–fiber (HC_In8)- vs. low–fiber (LC_In0)-purified diet (all B16-OVA tumors and anti–PD-1 treatment). FC, fold change. **D,** Venn diagram of significantly changed metabolites from the comparisons shown in **C**, showing minimal overlap between the high–fiber-purified diet and chow. For all panels, *n* = 7 to 8 mice per group.

Diet can influence the response to ICB by altering metabolism (24–26). Therefore, we next sought to understand how the addition of fiber to purified diets compares with chow metabolically (Fig. 1B). We conducted metabolomic analysis across three tumor models for serum (two for feces), including in the B16-OVA model that we used for metagenomics (See Supplementary Tables S5–S7 for complete serum metabolomics data). We first evaluated the overall relationship between all samples in each serum dataset using principal component analyses (PCA). In all three datasets, PCA revealed a clear separation along the first principal component (PC1), with a distinct cluster containing chow and another containing all purified diets, whereas fiber content within purified diets had a minimal impact (Fig. 2A; Supplementary Fig. S3A and S3B). The clustering was unaffected by whether the mice were treated with ICB (Fig. 2A; Supplementary Fig. S3A and S3B). These results indicate that diet type, i.e., chow or purified diet, has a far greater impact on driving metabolome changes than ICB or fiber content.

Similarly, in all serum datasets, hierarchical clustering of top significant metabolites was primarily driven by whether the diet was grain-based chow or purified (Fig. 2B; Supplementary Fig. S3C and S3D). For example, in the B16-OVA model, of 593 detected metabolites, 87 metabolites significantly changed between chow and low–fiber-purified diet (fold >2; FDR <0.05), whereas only 18 metabolites significantly changed between high–fiber- and low–fiber-purified diet, with much larger fold changes observed in the chow versus purified diet comparison (Fig. 2C). Moreover, the metabolite changes induced by chow and high–fiber-purified diets showed minimal overlap, with only two metabolites exhibiting the same directional change in both chow and high-fiber diets compared with the low–fiber-purified diet (Fig. 2D). Similar results were observed in the two other metabolomics datasets (Supplementary Table S8). These findings suggest that the chow-associated circulating metabolome is primarily driven by factors beyond fiber content.

We next analyzed the fecal metabolomics (see complete data in Supplementary Tables S9 and S10). More metabolites were significantly changed in feces than in serum for both chow versus purified diet and low-fiber versus high–fiber-purified diet (Supplementary Table S11). Nevertheless, similar to serum, PCA and hierarchal clustering showed that diet type (i.e., chow vs. purified) had a much larger influence than fiber content in driving fecal metabolite abundances (Supplementary Fig. S3E–S3H). Collectively, these data reveal that the metabolomics effects of diet processing (grain-based chow versus purified diet) exceed those of fiber content.

### Chow, but not isolated dietary fiber, favors ICB efficacy in the MC-38 colon carcinoma model

**Figure 3.**
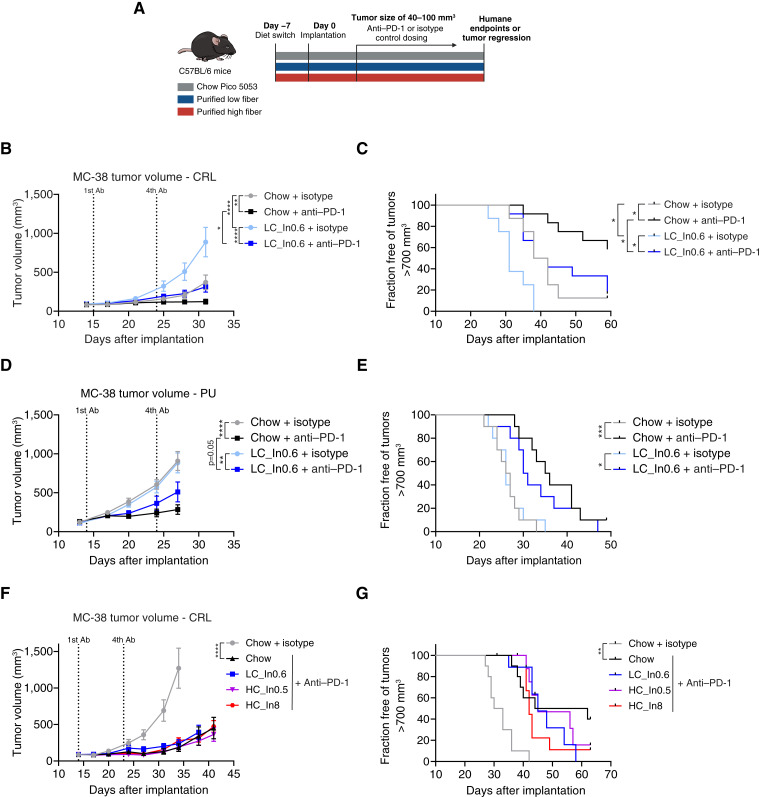
Grain-based chow favors ICB efficacy in MC-38 allografts, independently of dietary fiber. **A,** Study design for transplantable tumor models shown in Fig. 3 and 4. The first two MC-38 experiments (**B–E**) did not include a purified high-fiber dietary arm. Chow is intrinsically high fiber. **B** and **C,** Mean tumor volumes and fraction of mice free of tumors larger than 700 mm^3^ in mice implanted with subcutaneous MC-38 tumors, fed with chow or a low–fiber-purified diet, and treated with anti–PD-1 or isotype control in a study performed in CRL facilities. *n* = 8 for isotype control and *n* = 12 for anti–PD-1 groups. **D** and **E,** Same as in **B** and **C** but in a study performed in PU facilities. *n* = 10. **F** and **G,** Same as in **B** and **C** for mice fed with chow or purified diets with low or high insoluble fiber (cellulose, LC, or HC, respectively) and low or high soluble fiber (inulin). *n* = 10. *, *P* < 0.05; **, *P* < 0.01; ***, *P* < 0.001; ****, *P* < 0.0001, two-way ANOVA with the Tukey *post hoc* test at endpoint (day 38 for **F**) for **B**, **D**,** **and** F **andlog-rank test for **C**, **E**, and **G**. Values are mean ± SEM. (**A,** Created with sciencefigures.org.)

We next sought to determine the impact of dietary fiber on ICB efficacy in murine tumor models. We compared standard grain-based chow (high fiber) with purified diets with either low or high fiber (Fig. 3A). We were anticipating a pattern in which ICB efficacy is strong in chow and in the high–fiber-purified diet compared with low–fiber-purified diet. We first compared the effects of chow and a low–fiber-purified diet (LC, 0.6% inulin) on ICB efficacy in the MC-38 colon carcinoma allograft model in C57BL/6 mice. To assess the consistency across sites, the experiment was conducted both at CRL and PU. Despite somewhat lower calorie intake in mice on the low–fiber-purified diet, across both locations, body weight remained comparable among all groups throughout the study (Supplementary Fig. S4A–S4D). At Charles River, in accordance with Spencer and colleagues (12), chow feeding slowed tumor progression in isotype control groups and significantly improved ICB response compared with the low–fiber-purified diet (Fig. 3B and C). Chow-fed mice also showed a trend for a higher rate of complete tumor regression in response to ICB therapy (4/12 for chow vs. 2/12 for low–fiber-purified diet, Supplementary Fig. S4E). At Princeton, tumor growth in isotype control groups was comparable between the two diets. However, as seen at Charles River and in Spencer and colleagues (12), chow feeding improved ICB response, although the effect was only marginally significant (Fig. 3D and E), and no complete responders were observed in either group (Supplementary Fig. S4F). These results showed that grain-based chow tends to enhance ICB response in the MC-38 model at both sites.

Next, we aimed to determine the impact of individual fibers in purified diets on ICB response and the comparison between these diets and grain-based chow. To this end, we conducted another MC-38 study at Charles River. Mice were fed the same chow and low–fiber-purified diet or two high–fiber-purified diets with HC and either low or high inulin. Mice were treated with ICB (and for chow, also isotype control), and body weights and tumor volumes were monitored. Body weights were similar across all groups (Supplementary Fig. S4G). As a positive control, ICB treatment significantly suppressed tumor growth in chow-fed mice compared with isotype control. In this experiment, ICB efficacy in chow did not significantly differ from purified diet (Fig. 3F) although there was a trend toward improved overall survival (fraction free of tumors >700 mm^3^, Fig. 3G) and tumor-free survival in the chow group (2/10 for chow vs. 0/10 for low–fiber-purified diet, Supplementary Fig. S4H). Within purified diets, increasing levels of cellulose or inulin did not affect tumor growth in ICB-treated mice (Fig. 3F and G). Overall, our MC-38 studies suggest that grain-based chow modestly improves antitumor immunity compared with purified diets, whereas fiber addition does not.

### Purified cellulose, but not inulin or chow, improves ICB efficacy in one melanoma model, whereas diet has no effect in another

**Figure 4.**
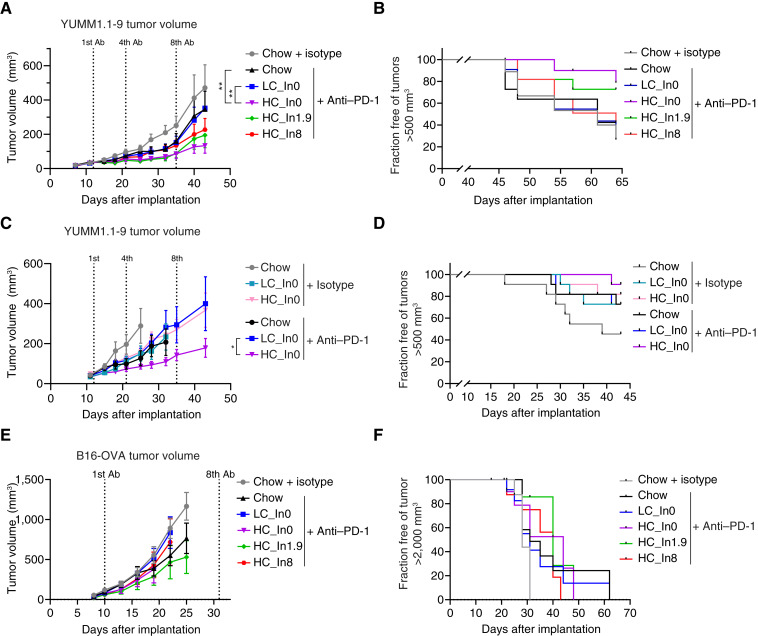
Purified cellulose enhances response to ICB therapy in YUMM1.1-9 but not in B16-OVA melanoma allografts. **A** and **B,** Mean tumor volumes and fraction of mice free of tumors larger than 500 mm^3^ for subcutaneous YUMM1.1-9 allografts in mice fed with chow or the indicated purified diets and treated with isotype control (for chow) or anti–PD-1. *n* = 9 to 11. **C** and **D,** Similar independent experiment for mice fed with chow, LC-, or HC-purified diets and treated with isotype control or anti–PD-1 antibody. *n* = 11. **E** and **F,** Mean tumor volumes and the fraction of mice free of tumors larger than 2,000 mm^3^ for subcutaneous B16-OVA allografts in mice fed chow or the indicated purified diets and treated with isotype control (for chow) or anti–PD-1. *n* = 7 to 12. *, *P* < 0.05; **, *P* < 0.01 for **A**, **C**, and **E** by a two-way ANOVA with the Tukey *post hoc* test at endpoint; values are mean ± SEM. For **B**, **D**, and **F**, log-rank test.

We next explored the effect of dietary fiber on ICB efficacy in melanoma allograft models. To better capture potential effects of soluble fiber, we used the more extreme version of low–soluble fiber diet we designed, switching from 0.6% to zero inulin. We further added HC diets with intermediate inulin (1.9%, matching the soluble fiber content in chow) and high inulin (8%). C57BL/6 mice were fed either chow or one of four purified diets and subcutaneously engrafted with YUMM1.1-9 melanoma cells (14). All groups were treated with ICB, whereas the chow-fed group also received an isotype control. As a positive control, ICB treatment suppressed tumor growth in chow-fed mice. Notably, there was no significant difference in efficacy between the extreme low–fiber-purified diet and chow (Fig. 4A and B). Intriguingly, however, purified diets high in cellulose improved ICB response compared with both chow and the LC-purified diet, with the strongest response observed in mice fed the HC/no inulin diet (Fig. 4A and B). These results suggest that insoluble fiber (cellulose), rather than soluble fiber (inulin), enhances ICB efficacy in this model.

Given the intriguing beneficial effect of insoluble fiber (cellulose) in this model, which seemed to be blunted by adding soluble fiber (inulin), we carried out an additional YUMM1.1-9 study comparing chow and soluble fiber–free LC- and HC-purified diets. Again in these studies, HC purified diet resulted in the best tumor control, both in the absence or presence of ICB therapy (Fig. 4C and D). Collectively, these studies suggest that, in the context of YUMM1.1-9 melanoma, purified diet high in insoluble fiber favors tumor control.

Using the same panel of diets in the B16-OVA subcutaneous melanoma model showed no significant differences in ICB efficacy across the diets (Fig. 4E and F), indicating that the impact of fiber on ICB can vary even in different tumors models of the same cancer type. For both melanoma models, food intake was lower in all purified diets relative to chow, with no major effects on body weights (Supplementary Fig. S5A–S5E). Taken together, these data indicate that cellulose, rather than soluble fiber, improves ICB responses in the YUMM1.1-9 melanoma model but not in B16-OVA.

### Cellulose tends to adversely affect ICB efficacy in the *Pold1*-mutant spontaneous tumor model

**Figure 5.**
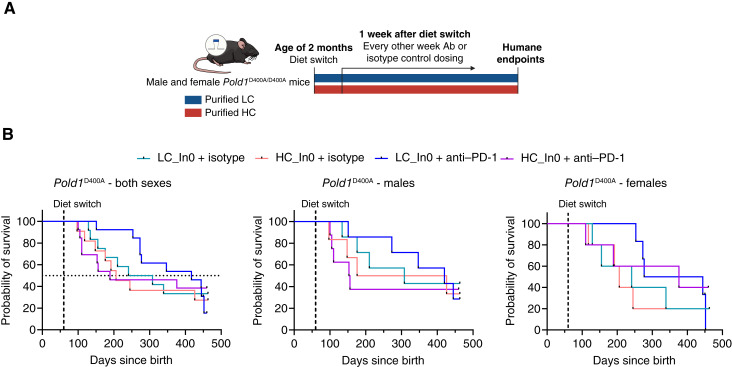
Dietary cellulose tends to negatively affect ICB efficacy in *Pold1*^D400A/D400A^ mice. **A,** Schematic showing *Pold1*^D400A/D400A^ study design. **B,** Survival curves of *Pold1*^D400A/D400A^ mice fed with the indicated HC or LC diets and treated with isotype control or anti–PD-1 antibody. Shown are plots for both sexes combined and males and females separately. *n* = 11 to 13, *n* = 6 to 8, and *n* = 5 to 6 mice per group for both sexes, males, and females, respectively. Overall survival curves are not significantly different. (**A,** Created with sciencefigures.org.)

Given the results from the YUMM1.1-9 model, we further investigated the effect of cellulose on ICB efficacy using *Pold1*^*D400A/D400A*^-mutant mice, a tumor-prone genetic model that we have recently developed that has a proofreading mutation in polymerase delta 1 and as a result has an elevated DNA mutation rate (mutator phenotype; bioRxiv 2024.06.10.597960). These mice spontaneously develop tumors in various organs and mostly succumb to thymic and splenic lymphomas before 1 year of age. Prophylactic ICB treatment delays cancer onset and extends median survival in these mice when fed a grain-based chow (bioRxiv 2024.06.10.597960). In contrast to the other mouse cancer models that examine dietary immune tumor control of existing tumors, this spontaneous tumor model also additionally addresses dietary immune impact on tumor initiation. To explore the role of cellulose, male and female *Pold1*^*D400A*^ mice were fed either LC or HC diets (both with no soluble fiber) starting at the age of ∼2 months, followed by biweekly ICB or isotype control treatments (Fig. 5A). Diet and ICB treatment did not affect body weight or food intake in either sex (Supplementary Fig. S6A–S6D). Interestingly, ICB treatment delayed early mortality in *Pold1*^*D400A*^ mice that were fed an LC rather than an HC diet (Fig. 5B, *P* < 0.05 for LC + anti–PD-1 vs. HC + anti–PD-1 for survival up to day 277, which corresponds to the 50th percentile of the total population’s survival). Thus, the effect of cellulose here is opposite to that in YUMM1.1-9 melanoma. In addition, median survival was substantially higher in the LC group treated with ICB (417 vs. 189 days for LC + anti–PD-1 and HC + anti–PD-1, respectively), but these differences did not reach statistical significance because of a greater proportion of late deaths in the LC + anti–PD-1 group (Fig. 5B). These results further indicate that the interaction between dietary fiber and ICB efficacy is specific to cancer type.

### High–fiber-purified diet, but not chow, modestly improves ICB efficacy in a genetically engineered breast cancer model

**Figure 6.**
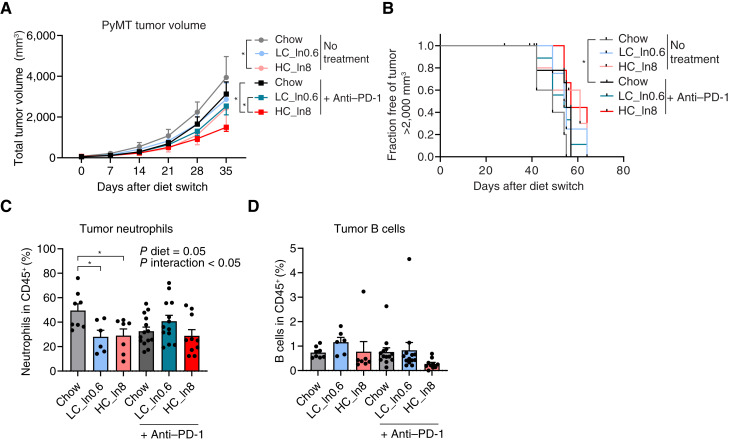
High–fiber-purified diet, but not chow, modestly enhances ICB efficacy in a breast cancer mouse model. **A,** Mean tumor volumes (calculated as the total volume of all individual mammary tumors per mouse) of MMTV-PyMT mice fed the indicated diets, with or without anti–PD-1 treatment. **B,** Fraction of mice free of a single primary tumor larger than 2,000 mm^3^. For **A** and **B**, *n* = 5 for isotype control and *n* = 10 for anti–PD-1 groups. **C** and **D,** Percentage of neutrophils and B cells among CD45^+^ cells in primary tumors collected at endpoint. *n* = 6 to 8 tumors collected from five mice for no treatment and 10 to 12 tumors from nine mice for anti–PD-1. *, *P* < 0.05. The log-rank test for **B** and an ANOVA with the Tukey *post hoc* test for other panels. Values are mean ± SEM.

We next sought to determine the effect of dietary fiber on ICB efficacy in breast cancer. We used MMTV-PyMT mice, a genetically induced breast cancer model that develops lung metastases. Mice were fed chow, low–fiber-purified diet (LC, 0.6% inulin), or high–fiber-purified diet (HC, 8% inulin) and were untreated or treated with an anti–PD-1 antibody. Mice fed with high-fiber diet gained slightly less body weight over the course of the study, and similar to the transplantable models, mice on purified diets had lower calorie intake (Supplementary Fig. S7A and S7B). Tumor progression in anti–PD-1–treated mice was slower in mice fed high–fiber-purified diet relative to both low–fiber-purified diet and chow diets (high fiber). The antitumor effect did not persist to result in a statistically significant survival benefit (Fig. 6A and B). Therefore, the combination of purified diet and dietary fiber modestly improves ICB efficacy in this model.

We next performed immune profiling in tumors collected at ethical endpoint (Supplementary Fig. S1A and S1B; Supplementary Table S12). Notably, although most immune cell populations measured were not altered, tumor-associated neutrophils, which can contribute to an immunosuppressive environment and promote tumor progression (27), were lower in mice untreated with anti–PD-1 in both purified diets relative to chow (Fig. 6C; Supplementary Fig. S7C). In addition, tumor B cells tended to be lower in anti–PD-1–treated mice fed with high–fiber-purified diet (Fig. 6D). Measuring lung metastases nodules at endpoint showed no significant differences between the groups (Supplementary Fig. S7D and S7E). Thus, in the MMTV-PyMT model, fiber modestly improves ICB response in the context of purified diets, but grain-based chow, despite being rich in fiber, does not show the same effect.

## Discussion

There is a great interest and potential in manipulating diet and/or microbiome to enhance immunotherapy. This is supported by clinical data from multiple trials that show correlations between diet or microbiome composition and ICB outcomes (28, 29). Specifically, fiber intake, as deduced from self-reported food diaries, correlated with improved clinical ICB outcomes (12). As fiber consumption likely correlates with many other factors, whether fiber itself drives ICB activity remains to be determined.

Previous mouse experiments compared grain-based chow (high fiber) to a low-fiber, high–fat-purified diet (12). This comparison was confounded by differing macronutrient composition (fat content) and diet purification (which affects a multitude of aspects beyond fiber including digestibility, phytochemicals, and minerals, which trump fiber in terms of its metabolomic impact). Here, we carried out controlled experiments examining the impact of fiber on ICB activity, finding no consistent role for fiber across five different mouse models.

Intriguingly, we observed consistent dietary effects in particular models. For example, across multiple experiments using MC-38 tumors at different sites, chow tended to result in better tumor growth control than low–fiber-purified diet, mirroring published results (12). The challenges were that (i) the benefits of chow were not replicated by adding fiber to the low–fiber-purified diet and (ii) trends did not hold across tumor types. For example, diet had no effect on tumor control in the B16-OVA model and high–fiber-purified diet (rather than chow) resulted in the best tumor control in the YUMM1.1-9 model. Thus, diet seems to affect immunologic tumor control in a context-dependent manner through dietary components beyond fiber. Our work was limited to murine tumor models and anti–PD-1 as the immunotherapy agent. We studied only a single insoluble fiber (cellulose) and soluble fiber (inulin). Different soluble fibers, such as pectins, β-glucans, and arabinoxylans, have distinct effects on the microbiome and their metabolic products (30, 31) could easily have differential effects on the immune system, which merit future study.

As opposed to this being a systematic study that applied the exact same dietary conditions across multiple models, experiments were designed sequentially to look for an affirmative effect of fiber (e.g., cellulose was tested in the *Pold1* model after it showed positive effects in the YUMM1.1-9 model). Each individual experiment was well controlled and appropriately powered (containing substantially more mice than the published affirmative results linking fiber to ICB efficacy; ref. 12), but heterogeneity in experimental designs complicates comparison across the five tumor models including two genetically engineered spontaneous tumor models. The data nevertheless establish the lack of a consistent positive effect in murine ICB of the tested dietary fibers.

This conclusion is important as it cautions against overly strong endorsement of dietary fiber intake *per se* for ICB patients until more data are available. Alternative guidance could be to eat a healthy plant-rich diet as such behavior could benefit patients even if the ultimate causative factor explaining the fiber–ICB correlation ends up being a different plant component. To this end, it would be valuable to re-examine existing clinical data for other potential correlates to ICB efficacy (e.g., plant foods in general; certain fruits, vegetables, grains, or legumes; and specific phytochemicals) and for future clinical efforts to take into account dietary complexity beyond fiber.

Patients undergoing immunotherapy are hungry for knowledge about foods to eat to maximize their chances of cancer remission. Our results argue for looking beyond the fibers (or at least beyond the commonly studied fibers of cellulose and inulin) and across a breadth of contexts, with the aim of ultimately providing the best possible guidance for future clinical evaluation and patient recommendations.

## Supplementary Material

Figure S1 Flow cytometry gating strategy

Figure S2 Extended microbiome analysis of mice on the different diets

Figure S3 Extended serum and fecal metabolomic analyses in the different tumor models

Figure S4 Extended data for the MC-38 tumor model

Figure S5 Extended data for the melanoma tumor models

Figure S6 Extended data for the Pold1 tumor model

Figure S7 Extended data for the PyMT tumor model

Table S1 Details of experimental diets

Table S2 Antibodies used for flow cytometry

Table S3 Gut microbiome taxonomic classification data

Table S4 Taxa exhibiting significant changes relative to the LC_In0 diet

Table S5 Serum metabolite peak areas from the B16-OVA model

Table S6 Serum metabolite peak areas from the YUMM1.1-9 model

Table S7 Serum metabolite peak areas from the PyMT model

Table S8 Significant serum metabolites from B16-OVA, YUMM1.1-9, and PyMT tumor models in chow or HC_In8 vs. LC_In0 comparisons

Table S9 Fecal metabolite peak areas from the B16-OVA model

Table S10 Fecal metabolite peak areas from the YUMM1.1-9 model

Table S11 Significant fecal metabolites from B16-OVA and YUMM1.1-9 tumor models in chow or HC_In8 vs. LC_In0 comparisons

Table S12 Tumor immune profiling in the PyMT model by flow cytometry
