# Supplementary material for: Dietary Fiber Lacks a Consistent Effect on Immune Checkpoint Blockade Efficacy Across Diverse Murine Tumor Models
Source: Cancer Res. 2025 Jun 20;85(17):3335–47. doi: 10.1158/0008-5472.CAN-24-4378 (PMC12402783; doi:10.1158/0008-5472.CAN-24-4378)
Supplement: Figure S1 — Flow cytometry gating strategy [file can-24-4378_figure_s1_suppsf1.pdf]

Supplementary Fig. 1

A

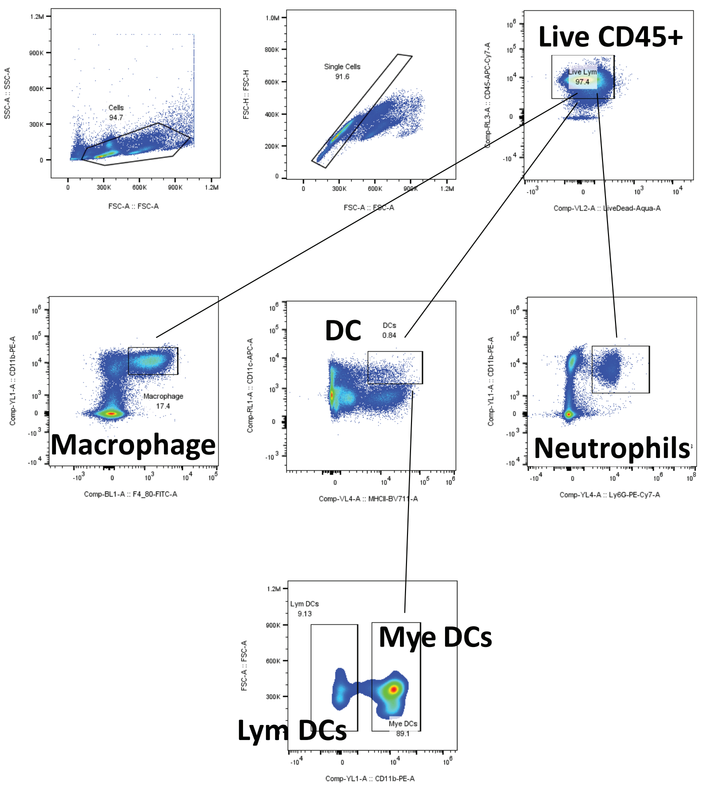

B

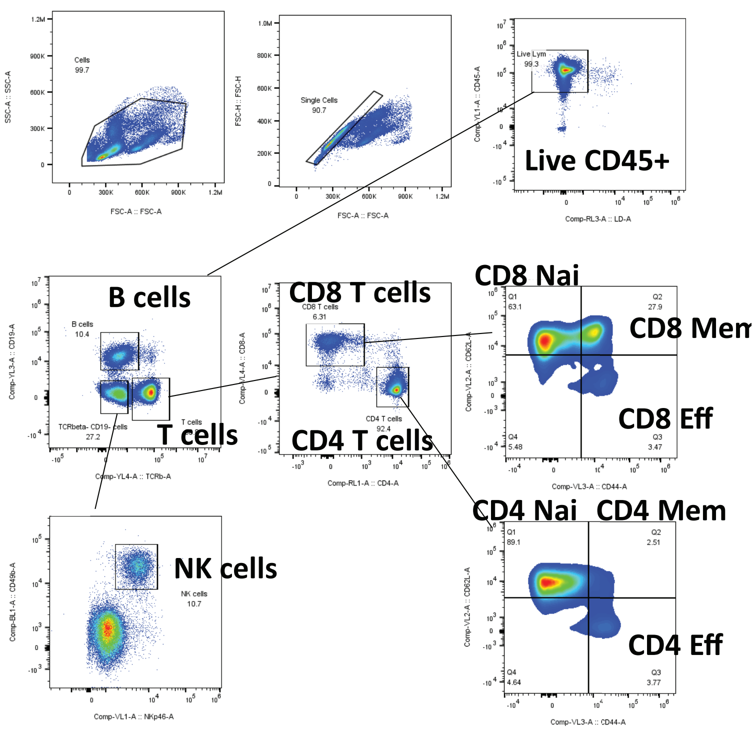

Supplementary Figure 1. A, Gating strategy for myeloid panel. Cells were first gated on singlet and live CD45$^+$ live lymphocytes. Then further gated on CD11b+F4/80+ macrophage, CD11b+Ly6G+ neutrophils. CD11c+MHCII+ DCs were further divided into Myeloid derived DCs (CD11b+ DCs) and Lymphoid derived DCs (CD11b- DCs). **B**, Gating strategy for lymphocyte panel. Cells were divided into B (CD19+ TCRb-) and T cells (CD19-TCRb+) after singlet/live CD45+ gate. NK cells were gated on CD19-TCRb-NKp46+CD49b+. T cells were divided into CD4 and CD8 T cells. The memory, effector and naïve state could be identified as CD62L+ CD44+, CD62L-CD44+, CD62L+CD44- respectively. See Supplementary Table S2 for list of antibodies used.
