# Supplementary material for: Dietary Fiber Lacks a Consistent Effect on Immune Checkpoint Blockade Efficacy Across Diverse Murine Tumor Models
Source: Cancer Res. 2025 Jun 20;85(17):3335–47. doi: 10.1158/0008-5472.CAN-24-4378 (PMC12402783; doi:10.1158/0008-5472.CAN-24-4378)
Supplement: Figure S2 — Extended microbiome analysis of mice on the different diets [file can-24-4378_figure_s2_suppsf2.pdf]

Supplementary Fig. 2

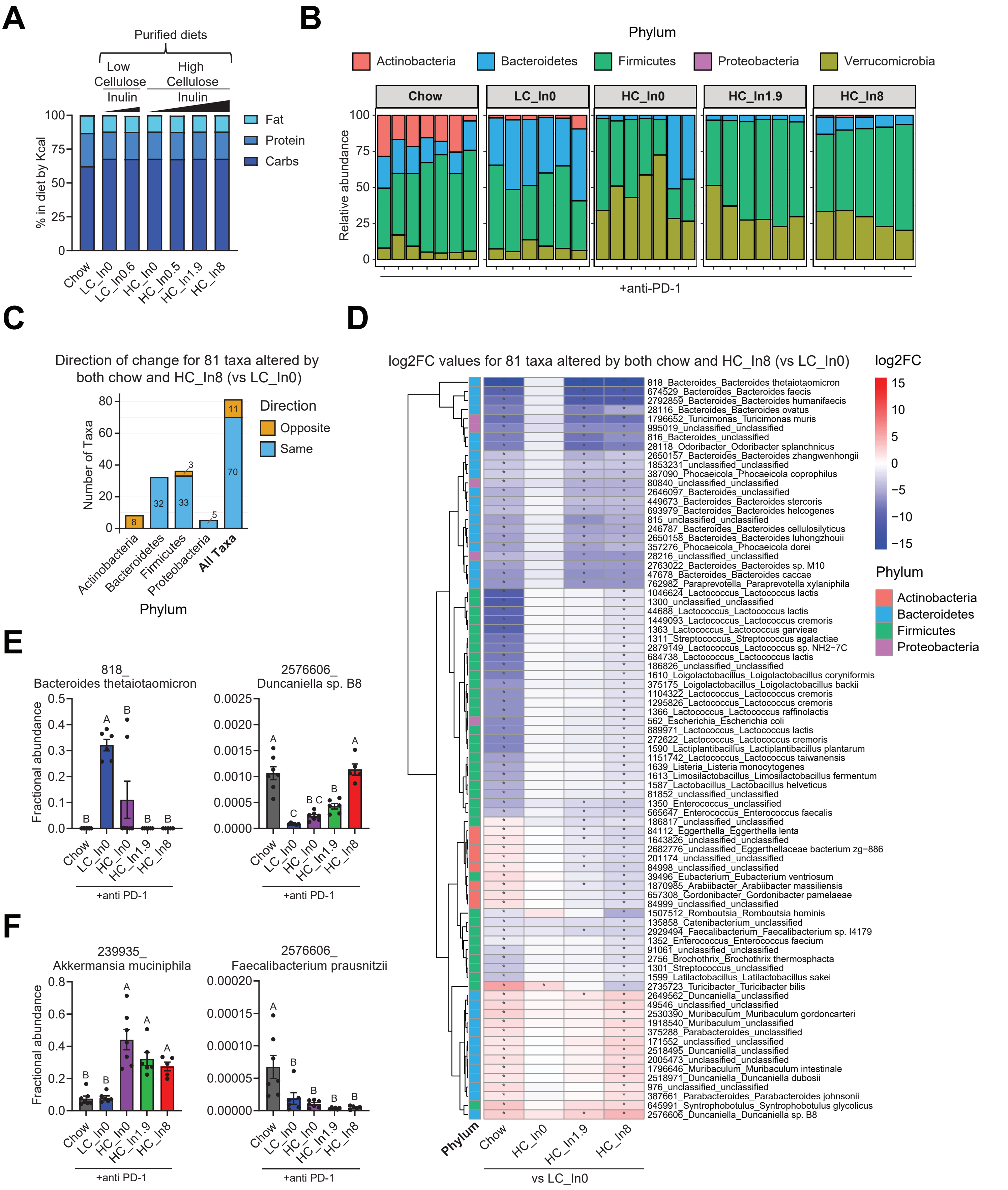

Supplementary Figure 2. A, Macronutrient composition of diets used in this study, represented by %Kcal. **B**, Relative abundances of microbiome composition at the phylum level, where each bar represents sample from an individual mouse. **C**, Direction of change for the 81 taxa significantly altered by both chow and the high-fiber purified diet (HC_In8) relative to low-fiber purified diet (LC_In0, all groups with anti-PD-1 treatment). With the exception of the *Actinobacteria* phylum, the vast majority—or all—taxa within each phylum showed consistent directional changes in response to both chow and the high-fiber purified diet. **D**, Heatmap showing the log2 fold change values for the 81 taxa altered by both chow and HC_In8 across the four indicated comparisons (all under anti-PD-1 treatment). Asterisks denote comparisons where changes are statistically significant (FDR < 0.01). **E**, Fractional abundances of two representative taxa from the top- and bottom-most sections of the heatmap, illustrating how high-fiber purified diets shift abundances toward levels observed in chow. **F**, Fractional abundances of two taxa previously associated with response to ICB. Differences are driven by high-fiber (cellulose) in purified diets for *A. muciniphila* and by chow for *F. prausnitzii*, but not by both. For panels E and F, values are mean ± SEM, with different letters above the bars indicate statistically significant differences between groups ($p < 0.05$) by one-way ANOVA followed by Tukey's post hoc test. For panels B-F, data are from n=5-7 mice per group.
