# Supplementary material for: Dietary Fiber Lacks a Consistent Effect on Immune Checkpoint Blockade Efficacy Across Diverse Murine Tumor Models
Source: Cancer Res. 2025 Jun 20;85(17):3335–47. doi: 10.1158/0008-5472.CAN-24-4378 (PMC12402783; doi:10.1158/0008-5472.CAN-24-4378)
Supplement: Figure S3 — Extended serum and fecal metabolomic analyses in the different tumor models [file can-24-4378_figure_s3_suppsf3.pdf]

Supplementary Fig. 3

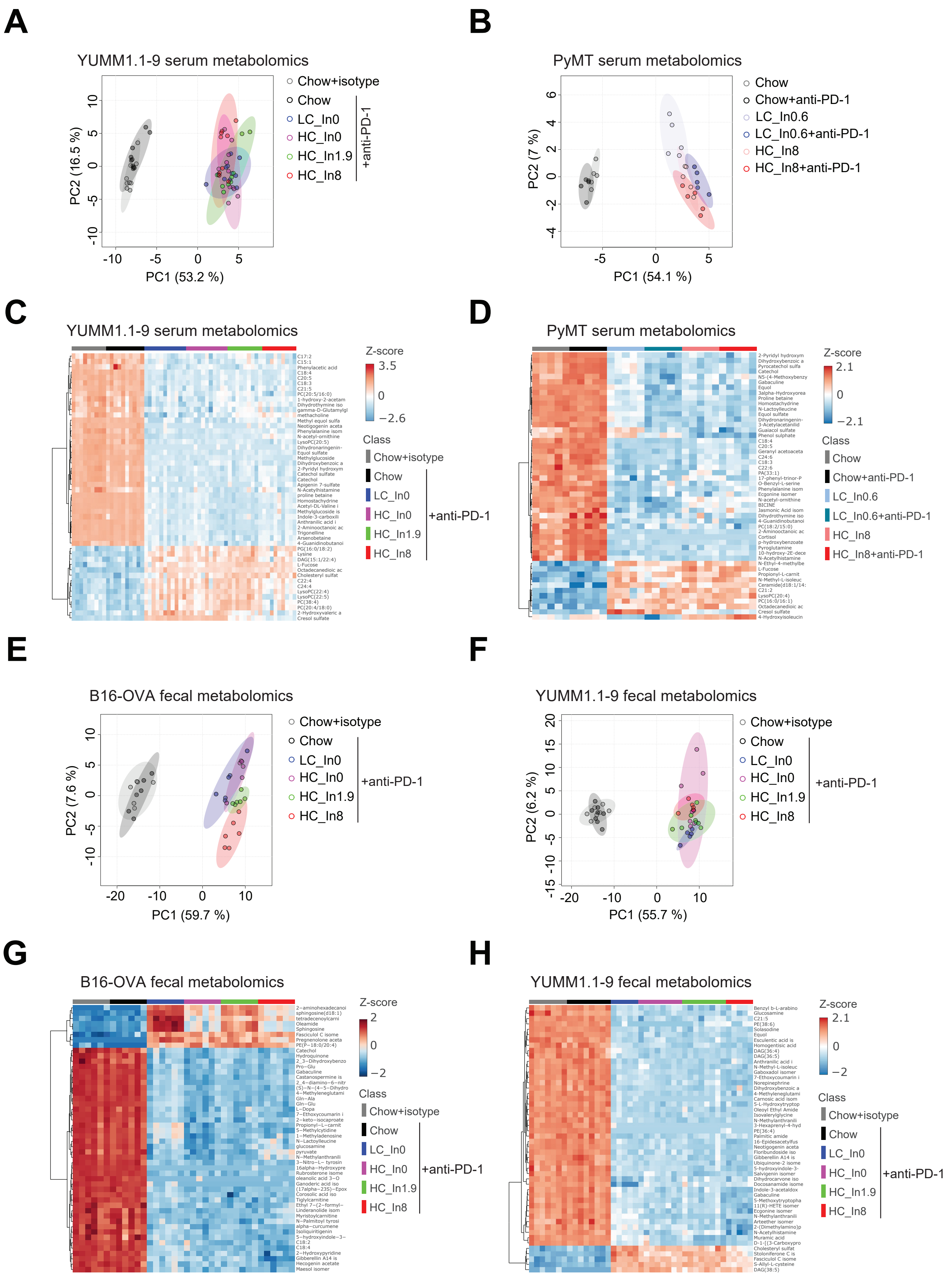

Supplementary Figure 3. A-B, Principal component analysis of serum metabolomics from YUMM1.1-9 and PyMT studies. **C-D**, Heatmap showing log-transformed relative abundances of top 50 metabolites by ANOVA from the YUMM1.1-9 and PyMT studies. For B-E, *n*=9-11 mice per group for YUMM1.1-9 and *n*=5 mice per group for PyMT. **E-F**, Principal component analysis of fecal metabolomics from the B16-OVA and YUMM1.1-9 studies. **G-H**, Heatmap showing log-transformed relative abundances of top 50 metabolites by ANOVA from the B16-OVA and YUMM1.1-9 studies. For F-I, *n*=6 mice per group for B16-OVA and *n*=5-8 mice per group for YUMM1.1-9
