# Supplementary material for: Dietary Fiber Lacks a Consistent Effect on Immune Checkpoint Blockade Efficacy Across Diverse Murine Tumor Models
Source: Cancer Res. 2025 Jun 20;85(17):3335–47. doi: 10.1158/0008-5472.CAN-24-4378 (PMC12402783; doi:10.1158/0008-5472.CAN-24-4378)
Supplement: Figure S4 — Extended data for the MC-38 tumor model [file can-24-4378_figure_s4_suppsf4.pdf]

Supplementary Fig. 4

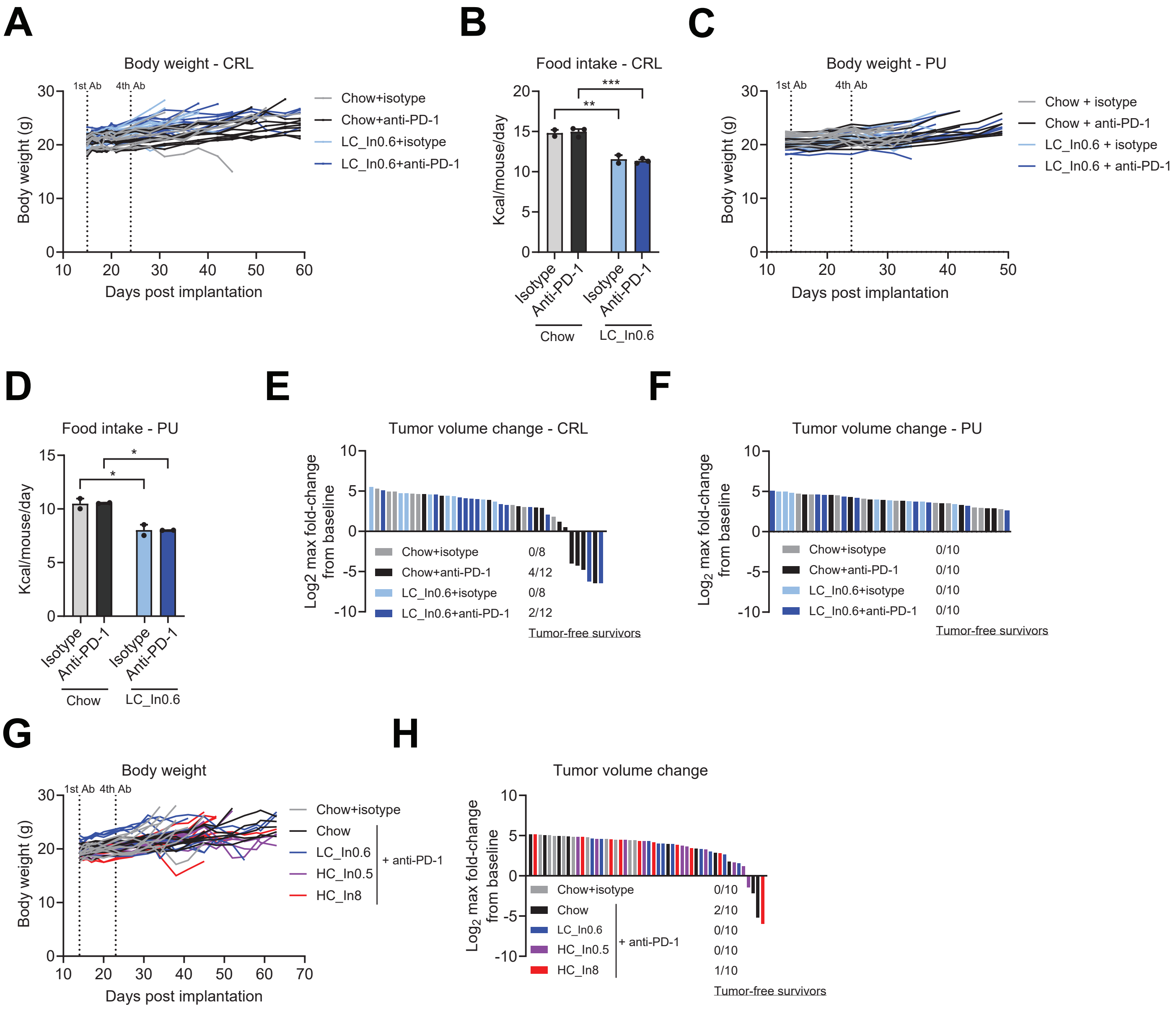

Supplementary Figure 4. **A-B**, Body weights of individual mice and food intake averaged from 2-3 cages per group for the MC-38 experiment performed at Charles River Laboratories (CRL). Values in B are mean ±SEM. $n = 8$ for isotype control and $n = 12$ for anti PD-1 groups. **C-D**, Same, for the MC-38 experiment performed at Princeton University (PU). $n = 10$, food intake averaged from 2-3 cages per group. Values in D are mean ±SEM. **E**, Maximal tumor volume change from baseline, with the number of tumor-free survivors for each group indicated, in the experiment performed at CRL. $n = 8$ for isotype control and $n = 12$ for anti PD-1 groups. Panels A, B and E correspond to the study presented in main text Figures 3B and C. **F**, Same, for the experiment performed at PU. $n = 10$. Panels C, D and F correspond to the study presented in main text Figures 3D and E. **G-H**, Body weights of individual mice and maximal tumor volume change from baseline with tumor free survivors indicated, corresponding to the study presented in Figures 3F and G of the main text. $n = 10$.
