# Supplementary material for: Dietary Fiber Lacks a Consistent Effect on Immune Checkpoint Blockade Efficacy Across Diverse Murine Tumor Models
Source: Cancer Res. 2025 Jun 20;85(17):3335–47. doi: 10.1158/0008-5472.CAN-24-4378 (PMC12402783; doi:10.1158/0008-5472.CAN-24-4378)
Supplement: Figure S5 — Extended data for the melanoma tumor models [file can-24-4378_figure_s5_suppsf5.pdf]

Supplementary Fig. 5

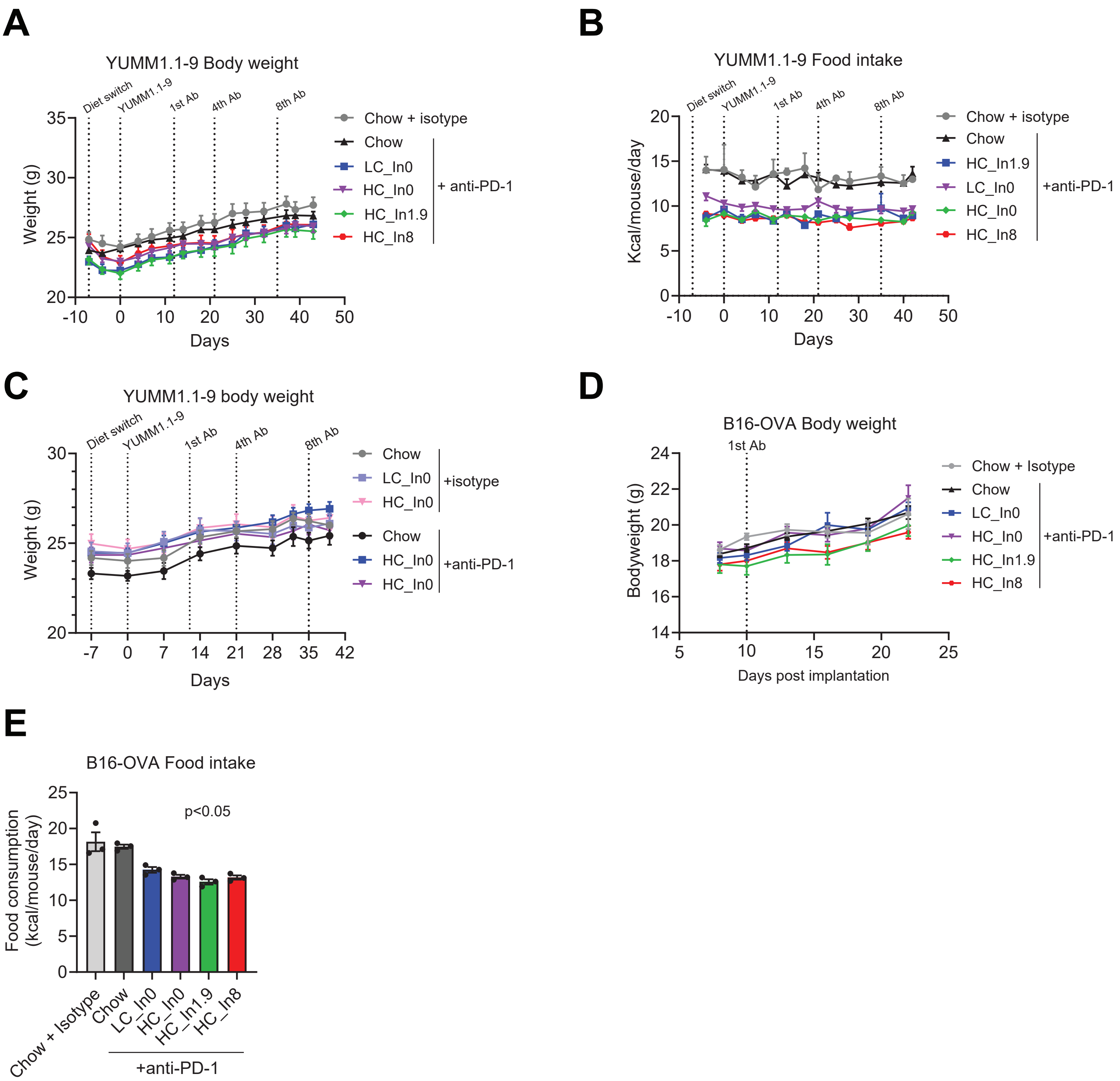

Supplementary Figure 5. **A-B**, Mean body weight and food intake of mice bearing subcutaneous YUMM1.1-9 allografts from the experiment shown in Figures 4A and B of the main text. n=9-11 for A, n=2-3 cages for B. p<0.05 for A and p<0.0001 for B by two-way ANOVA for main diet effect. **C**, Mean body weights of mice bearing subcutaneous YUMM1.1-9 allografts from the experiment presented in main text Figures 4C and D. n = 11. **D-E**, Mean body weight and food intake of mice bearing subcutaneous B16-OVA allografts from the experiment presented in main text Figures 4E and F. n=7-12 for D, n=3 cages for E. p<0.05 for E by one-way ANOVA. For all panels, error bars indicate SEMs.
