# Supplementary material for: Dietary Fiber Lacks a Consistent Effect on Immune Checkpoint Blockade Efficacy Across Diverse Murine Tumor Models
Source: Cancer Res. 2025 Jun 20;85(17):3335–47. doi: 10.1158/0008-5472.CAN-24-4378 (PMC12402783; doi:10.1158/0008-5472.CAN-24-4378)
Supplement: Figure S6 — Extended data for the Pold1 tumor model [file can-24-4378_figure_s6_suppsf6.pdf]

# Supplementary Fig. 6

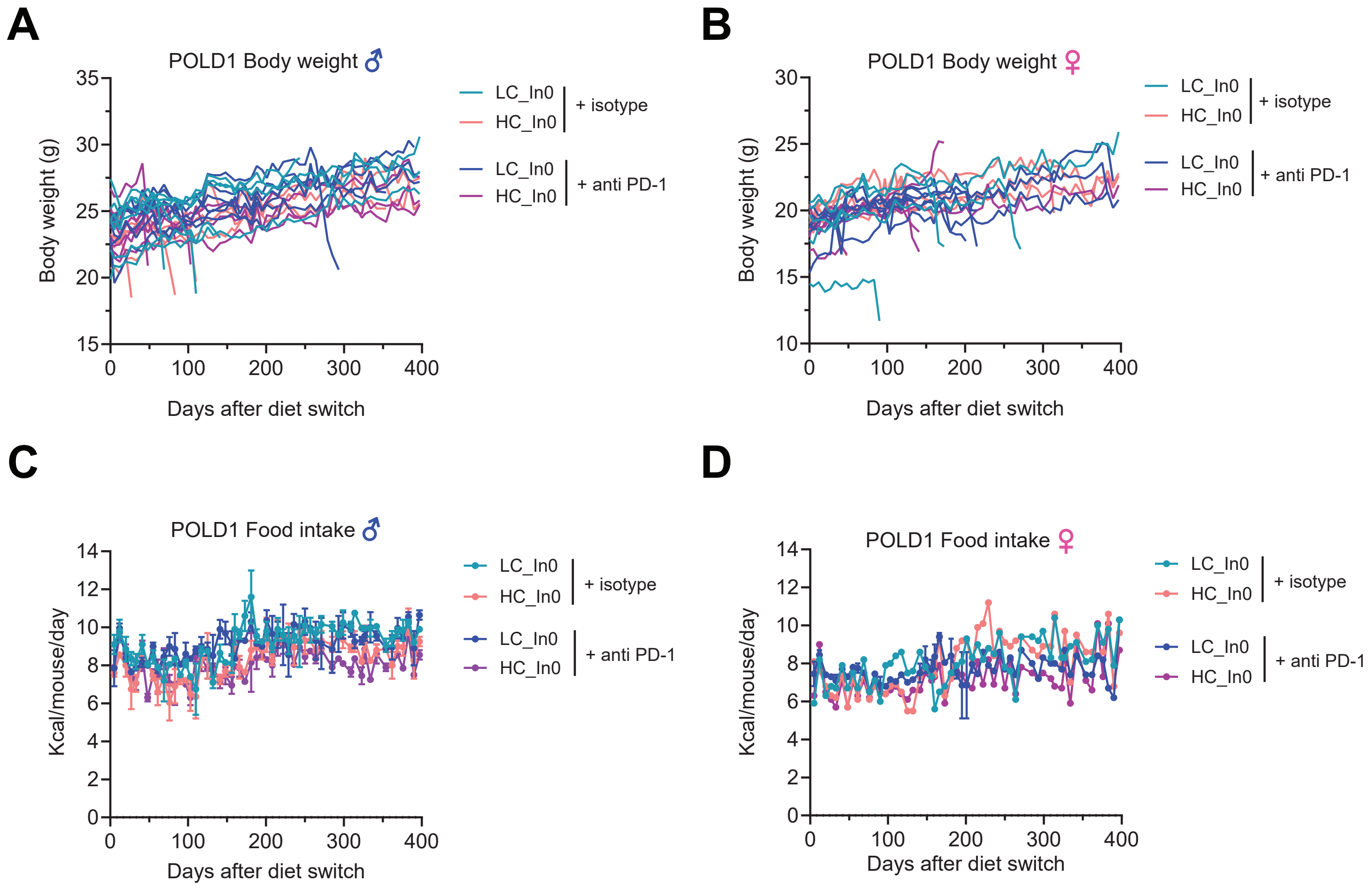

**Supplementary Figure 6**. **A-B**, Body weights of Pold1$^{D400A/D400A}$ male and female mice receiving high or low cellulose diet and treated with anti-PD-1 or isotype control antibody. *n*=6-8 for males, *n*=5-6 for females. **C-D**, Food intake of the same mice. *n*=2 cages for males, *n*=1-2 cages for females. Values are mean ± SEM.
