# Supplementary material for: Dietary Fiber Lacks a Consistent Effect on Immune Checkpoint Blockade Efficacy Across Diverse Murine Tumor Models
Source: Cancer Res. 2025 Jun 20;85(17):3335–47. doi: 10.1158/0008-5472.CAN-24-4378 (PMC12402783; doi:10.1158/0008-5472.CAN-24-4378)
Supplement: Figure S7 — Extended data for the PyMT tumor model [file can-24-4378_figure_s7_suppsf7.pdf]

Supplementary Fig. 7

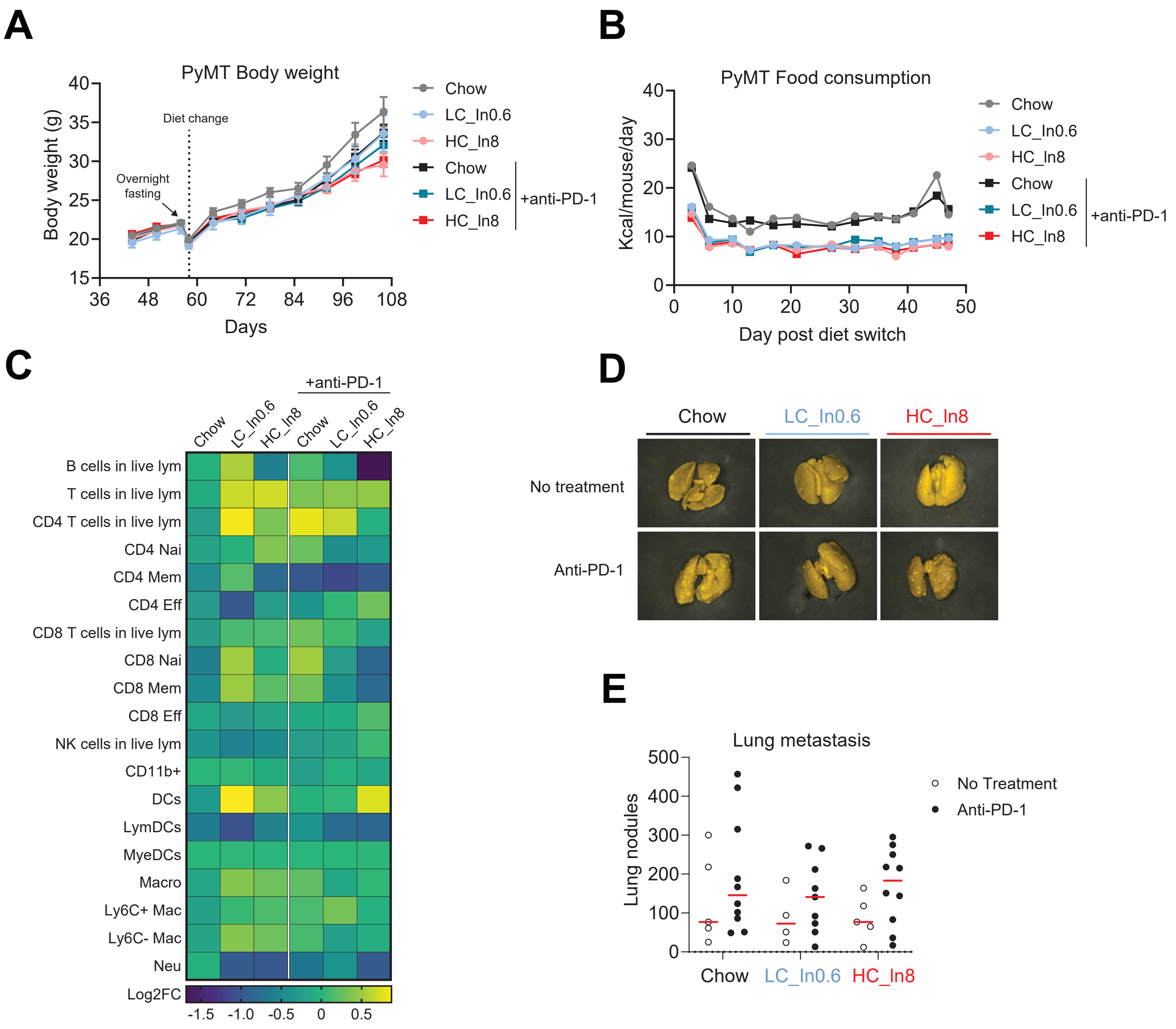

Supplementary Figure 7. A-B, Mean body weight (± SEM) and mean food intake of MMTV-PyMT mice from the experiment presented in main text Figure 6A and B. *n*=5 for isotype control and *n*=10 for anti PD-1 groups for panel A, *n*=1-2 cages for panel B. **C,** Heatmap showing the percentage of immune cell populations in primary tumors relative to the chow no-treatment group. Each square represents the average of *n*=6-8 tumors collected from 5 mice for no treatment and 10-12 tumors from 9 mice for anti PD-1. See Supplementary Table S11 for raw data. **D-E**, Representative images and quantification of lung metastases at humane endpoints from the experiment presented in main text Figure 6A and B. *n*=4-5 for no treatment and *n*=9-10 for anti PD-1 groups.
